# Higher systemic immune-inflammation index and systemic inflammation response index levels are associated with stroke prevalence in the asthmatic population: a cross-sectional analysis of the NHANES 1999-2018

**DOI:** 10.3389/fimmu.2023.1191130

**Published:** 2023-08-04

**Authors:** Wenke Cheng, Xiancong Bu, Chunhua Xu, Grace Wen, Fanliang Kong, Huachun Pan, Shumin Yang, Siwei Chen

**Affiliations:** ^1^ Medical Faculty, University of Leipzig, Leipzig, Germany; ^2^ Department of Neurology, Zaozhuang Municipal Hospital, Shandong, China; ^3^ Department of Recuperation, Lintong Rehabilitation and Recuperation Center, Shanxi, China; ^4^ University Medical Center of Göttingen, Georg-August University, Göttingen, Germany; ^5^ College of Veterinary Medicine, Huazhong Agricultural University, Wuhan, China; ^6^ State Key Laboratory of Agriculture Microbiology, College of Veterinary Medicine, Huazhong Agricultural University, Wuhan, China; ^7^ Department of Cardiovascular Medicine, Nanchang People's Hospital (The Third Hospital of Nanchang), Jiangxi, China

**Keywords:** systemic immune-inflammation index, national health and nutrition examination survey, stroke, asthma, cross-sectional, systemic inflammation response index

## Abstract

**Background:**

Significant evidence suggests that asthma might originate from low-grade systemic inflammation. Previous studies have established a positive association between the systemic immune-inflammation index (SII) and the systemic inflammation response index (SIRI) levels and the risk of stroke. However, it remains unclear whether SII, SIRI and the prevalence of stroke are related in individuals with asthma.

**Methods:**

The present cross-sectional study used data from the National Health and Nutrition Examination Survey (NHANES) conducted between 1999 and 2018. SII was calculated using the following formula: (platelet count **×** neutrophil count)**/**lymphocyte count. SIRI was calculated using the following formula: (neutrophil count × monocyte count)/lymphocyte count. The Spearman rank correlation coefficient was used to determine any correlation between SII, SIRI, and the baseline characteristics. Survey-weighted logistic regression was employed to calculate odds ratios (ORs) and 95% confidence intervals (CIs) to determine the association between SII, SIRI, and stroke prevalence. The predictive value of SII and SIRI for stroke prevalence was assessed through receiver operating characteristic (ROC) curve analysis, with the area under the ROC curve (AUC) being indicative of its predictive value. Additionally, clinical models including SIRI, coronary heart disease, hypertension, age, and poverty income ratio were constructed to evaluate their clinical applicability.

**Results:**

Between 1999 and 2018, 5,907 NHANES participants with asthma were identified, of which 199 participants experienced a stroke, while the remaining 5,708 participants had not. Spearman rank correlation analysis indicated that neither SII nor SIRI levels exhibited any significant correlation with the baseline characteristics of the participants (r<0.1). ROC curves were used to determine the optimal cut-off values for SII and SIRI levels to classify participants into low- and high-level groups. Higher SII and SIRI levels were associated with a higher prevalence of stroke, with ORs of 1.80 (95% CI, 1.18-2.76) and 2.23 (95% CI, 1.39-3.57), respectively. The predictive value of SIRI (AUC=0.618) for stroke prevalence was superior to that of SII (AUC=0.552). Furthermore, the clinical model demonstrated good predictive value (AUC=0.825), with a sensitivity of 67.1% and specificity of 87.7%.

**Conclusion:**

In asthmatics, higher levels of SII and SIRI significantly increased the prevalence of stroke, with its association being more pronounced in individuals with coexisting obesity and hyperlipidaemia. SII and SIRI are relatively stable novel inflammatory markers in the asthmatic population, with SIRI having a better predictive value for stroke prevalence than SII.

## Introduction

Asthma is a common disease affecting children and adults and is associated with high morbidity, mortality, and economic burden worldwide (1). Asthma is responsible for approximately 495,000 deaths yearly (2). The disease is characterised by chronic airway inflammation, airway hyperreactivity, and reversible airflow limitation, which can lead to complications and comorbidities as lung function declines (3). As a result, emergency medical care is often required due to asthma-related complications (3). Although asthma is not entirely curable, ongoing assessment of symptoms and the risk of future adverse outcomes is necessary (1). Patients with poorly controlled asthma and frequent exacerbations are at an increased risk for cardiovascular disease and ischaemic stroke (4, 5). Studies have demonstrated that respiratory diseases, including asthma, are associated with a higher incidence of stroke, suggesting a significant interaction between these conditions and that asthma might be an independent risk factor for stroke (6). In addition, the severity of asthma is linearly related to the development of stroke (7), emphasising the importance of the interaction between the central nervous system (CNS) and the respiratory system (8).

Inflammation is a complex physiological response aimed at repairing tissue damage, involving cells, plasma components, and cellular products (9). This response can occur in any vascular compartment of the body, including the CNS. Inflammation is a primary factor in the acute progression of brain stroke, where cerebral ischaemia triggers a strong inflammatory response, resulting in peripheral leukocyte infiltration into the brain parenchyma and endogenous microglial cell activation (10, 11). Cessation of cerebral blood flow results in energy depletion and necrotic neuronal death, thereby triggering an immune response and ultimately resulting in inflammatory cell activation and infiltration (11). Similar to stroke, it is now recognised that low-grade systemic inflammation is a feature of chronic asthma, extending beyond the airways (12–14). Asthma also has a systemic impact, contributing to the development of atherosclerosis (15) and leading to measurable changes in vascular structure and function (13, 16, 17). This might be due to airway remodelling caused by the inflammatory response and its irreversible airway obstruction (18), leading to a decline in lung function, chronic hypoxia in the body, and oxidative stress. Asthmatic patients are also known to present with a hypercoagulable state (15). These findings suggest that inflammation is a common pathophysiological process in the progression of asthma and brain tissue diseases, and there is a significant interplay between asthma and stroke.

While inflammation is a physiological process for repairing damaged tissues, its uncontrolled progression can result in tissue damage. Laboratory tests and indices are emerging to better understand an individual’s inflammatory status. The search for biomarkers to evaluate airway inflammation has garnered increasing attention, with new inflammatory biomarkers, such as neutrophil-lymphocyte ratio (NLR) and platelet-lymphocyte ratio (PLR) being developed. Previous studies have demonstrated that high NLR and PLR are strongly associated with adverse cardiovascular disease, malignancy, and chronic kidney disease (19–23). In contrast to NLR and PLR, SII and SIRI are newly defined, easily accessible, and objective indices comprising four haematological lineages, namely neutrophils, monocytes, lymphocytes, and platelets, which provide more clinical information than the two peripheral blood cells (14). The four cell lineages used in calculating SII and SIRI play a crucial role in asthma pathogenesis. Neutrophils, the most abundant white blood cells, play a crucial role in initiating innate immune responses, working together with monocytes (24). Studies have revealed that symptomatic asthmatics experience elevated and activated levels of peripheral blood neutrophils (25). These levels decrease following symptom relief or treatment but remain higher than healthy levels. Neutrophils can also secrete chemotactic factors and preformed granule proteins, attracting monocytes/macrophages to the site of infection, resulting in immune infiltration (26). Monocytes not only contribute to airway remodelling and local inflammation in patients with asthma but also release various pro-inflammatory factors, such as tumour necrosis factor (TNF-α) and interleukins (ILs) (27). Moreover, evidence suggests that patients with asthma have an increased number of activated monocytes in their peripheral blood, indicating their direct involvement in the immunopathology of asthma (28). Lymphocytes are indicators of allergic inflammation regulators (29), and their relative decrease in patients with asthma could be due to the increase in neutrophil and monocyte numbers. During airway inflammation, lymphocytes might infiltrate from the blood into the airway tissues, resulting in a relative decrease in blood lymphocyte numbers. Furthermore, immunosuppressive drugs (such as corticosteroids) might inhibit lymphocyte proliferation and activity. It has been recently shown that platelets interact with different types of leukocytes during inflammation and bind to different immune cells by altering their surface expression and releasing granule contents (24). Platelets mediate immune and inflammatory responses and might serve as potential targets for treating asthma. The interaction between platelets and neutrophils is crucial for initiating immune responses. In asthma patients, there is often a hypercoagulable state characterised by increased platelet count (15). When exposed to certain infectious or inflammatory stimuli, platelets alter the expression of P-selectin or CD40 on their surface, allowing them to interact with circulating leukocytes (30). These leukocytes recognise platelet p-selectin or CD40 via white blood cell P-selectin glycoprotein ligand 1 and CD154, respectively, resulting in the formation of platelet-white blood cell aggregates (24). Such interactions promote innate immune responses, primarily by forming aggregates with circulating monocytes or neutrophils (30). Studies in animal models have confirmed that platelet P-selectin is a necessary condition for lymphocyte recruitment (31). Thus, SII and SIRI serve as superior indicators of the equilibrium between inflammation and immune responses compared with other systemic inflammation markers. In asthmatic populations, the relative increase in neutrophil, monocyte and platelet counts and the relative decrease in lymphocytes can disrupt the balance between the normal immune and inflammatory systems. Although prior research has established a positive correlation between SII and SIRI levels and stroke risk (32–34), their relationship with stroke in the asthmatic population remains unknown. Therefore, this study aimed to investigate the relationship between SII and SIRI levels and the prevalence of stroke in the asthmatic population.

## Methods

### Study design and population

This study used a cross-sectional design, and its data were obtained from the National Health and Nutrition Examination Survey (NHANES) conducted from 1999 to 2018. NHANES is a nationally representative survey of the United States civilian non-institutionalised population, and it employed a complex stratified multistage probability design that involved interviews, physical examinations at home or mobile examination centres (MECs), and laboratory tests. The survey is conducted biennially, and its detailed sampling and data collection procedures have been previously published (35). NHANES was conducted by the National Centre for Health Statistics of the Centres for Disease Control and Prevention and was approved by the NHANES Institutional Review Board (36). Written informed consent was obtained from all participants.

NHANES is a publicly accessible database, except for Limited Access Data. For this study, data were obtained from the publicly accessible portion of NHANES and were used in accordance with the relevant data usage regulations. Furthermore, all personal information of the participants was anonymised to protect their rights.

### Inclusion and exclusion criteria

Adults with asthma, aged ≥18 years were included in the study.

The exclusion criteria were as follows:

1. Those who were aged<18 years and did not have asthma.
2. Participants without weighted data.
3. Participants who were pregnant or had cancer.
4. Participants without SII and SIRI data.
5. Participants with chronic obstructive pulmonary disease (COPD).

### Definition of SII

Complete blood cell count measurements of blood specimens were detected on MECs automated analytical instrument (Beckman Coulter MAXM; Beckman Coulter Inc.). Lymphocyte, neutrophil, monocyte, and platelet counts were expressed in ×10^3^ cells/μml.

The following formula was used to calculate SII and SIRI (37, 38):


SII = (platelet count × neutrophil count)/lymphocyte count

SIRI = (neutrophil count × monocyte count)/lymphocyte count

### Diagnosis of asthma and stroke

Asthma was diagnosed based on self-administered questionnaires completed by the participants during the clinic visit. The questionnaires comprised two questions: 1) “Has a doctor or other healthcare professional ever told you that you have asthma”? and 2) “Have you had wheezing or whistling in your chest in the past 12 months”? Those who responded positively to both questions were classified as having asthma. Non-responders who did not have a history of smoking, chronic bronchitis, or emphysema, but used anti-asthmatic medications were also classified as having asthma.

Similarly, stroke was diagnosed based on self-reported medical history from the Medical Conditions section of NHANES. Specifically, participants were asked, “Has a physician or other health professional ever told you that you had a stroke”? Depending on their response to this self-reported question, participants were classified into either the “stroke” or the “non-stroke” group.

### Other variables of interest

Information on participants’ age, gender race, education level, household income, smoking and drinking status, medical history, and medication use was collected using a standardised questionnaire. Self-reported medical history was based on previous medical records from healthcare professionals or physicians. Biochemical parameters were measured using strict procedures described in the NHANES Laboratory/Medical Technician Procedures Manual (39).

The following variables were further categorised to facilitate data integration:

1. Race: non-Hispanic white individuals, non-Hispanic black individuals, Mexican-American individuals, or other races.
2. Educational level: less than the ninth grade, 9^th^-11^th^ grade/high school or equivalent, or college graduate or above.
3. Smoking status: active smoker (>100 cigarettes/lifetime and currently smoking on certain days or every day) or non-active smoker (including never [<100 cigarettes/lifetime] or former smoker [100 cigarettes/lifetime and absolutely no smoking now]) (40).
4. Drinking status: active alcohol consumer (including current light/moderate drinker [ ≤ 1 drink/day for women and ≤2 drinks/day for men in the past year] or current heavy drinker [>1 drink/day for women and >2 drinks/day for men in the past year]) and non-active alcohol user (including never [<12 drinks/lifetime] and former drinker [≥12 drinks/lifetime but not in the past year]) (41).
5. Considering the differences between asthma treatments between generations, we divided the participants into subgroups 1999-20004, 2005-2010, 2011-2018.

### Statistical analysis

Appropriate weights (MECs weights) were applied to the data to account for oversampling, nonresponse, and noncoverage and provide nationally representative estimates. The weighting methodology is described in detail on the NHANES website (42).

Baseline demographic characteristics are presented as means (standard error) for continuous variables and weighted percentages (95% confidence interval, 95% CI) for categorical variables. Participants were divided into the stroke and non-stroke groups based on their stroke history. As most data were skewed, continuous and categorical variables were compared between the two groups using the weighted Mann–Whitney and chi-square tests, respectively. The Spearman rank correlation coefficient was used to assess any association between SII, SIRI, and baseline characteristics. A correlation coefficient (r) of<0.1 indicated no correlation, 0.1-0.3 indicated low correlation, 0.4-0.6 indicated moderate correlation, and 0.7-1.0 indicated significant correlation (43). The optimal cut-off values for SII and SIRI were determined using the receiver operating characteristic (ROC) curve. The area under the curve (AUC) was calculated to assess the model’s predictive power. Logistic regression models were employed to estimate odds ratios (ORs) and 95% CIs between SII and SIRI levels (low vs. high) and stroke prevalence.

The study conducted subgroup analyses by stratifying participants based on clinical characteristics, including gender (male or female), age (<60 years or ≥60 years), body mass index (BMI) (<30 kg/m^2^ or ≥30 kg/m^2^), race (non-Hispanic white individuals, non-Hispanic black individuals, Mexican-American individuals, and other race individuals), coronary heart disease (no/yes), diabetes mellitus (no/yes), hypertension (no/yes), hyperlipidaemia (no/yes) and the year of entry (1999-20004, 2005-2010, 2011-2018),. The study also obtained P-values for interaction in these groups.

Furthermore, a clinical model was constructed using multivariate logistic regression models to identify variables and develop nomograms for predicting the risk of stroke prevalence. Each included variable was assigned a point value on the top line of the total score based on its regression coefficient. The total score was calculated by summing the scores of all variables for each patient. The relationship between the total score and the prognosis was presented at the bottom of the nomogram.

R (http://www.R-project.org, R Foundation) and EmpowerStats (version 4.2.0, www.R-project.org, X&Y Solutions, Inc., Boston, MA) were used to perform all analyses. Statistical significance was set at P<0.05(two-tailed).

## Results

**Figure 1:**
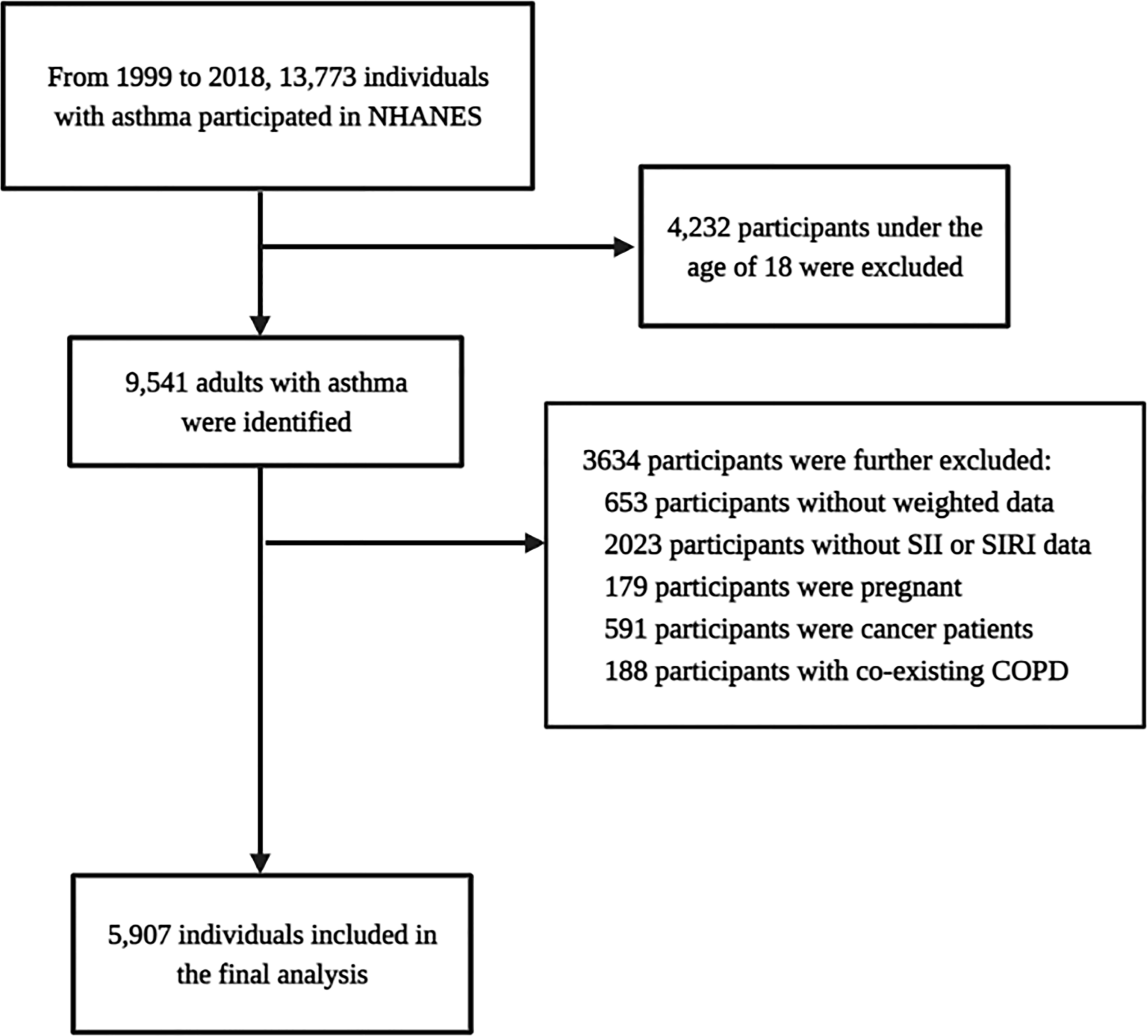
The study flow.

A total of 13,773 individuals with asthma participated in the NHANES survey between 1999 and 2018. Among them, 4,232 were excluded, as they were younger than 18 years. Among the remaining 9,541 participants, 3,634 were further excluded for the following reasons: 1) a total of 653 participants had no weight data, 2) SII data was missing for 2,023 participants, 3) A total of 179 participants were pregnant, 4) cancer was diagnosed in 591 participants, and 5) COPD was diagnosed in 188 participants. Finally, 5,907 individuals with asthma were included in the analysis, as presented in **Figure 1**. Of these, 199 participants had a history of stroke, while 5,708 did not. This represents 546,572 and 22,124,799 individuals, respectively. The stroke population had a higher mean age of 63.44 years, as presented in **Table 1**. Compared with the non-stroke population, the stroke population had a lower poverty-income ratio (PIR) and estimated glomerular filtration rate (eGFR) and a higher prevalence of diabetes mellitus, hypertension, coronary heart disease, and hyperlipidaemia (P<0.05).

**Table 1:** Survey-weighted baseline characteristics of individuals with asthma from NHANES 1999 to 2018 (Stroke N=199, representing 546,572 individuals; non-Stroke N=5,708, representing 22,124,799 individuals).

	non-Stroke (N=5708)	Stroke (N=199)	P-value
Age	43.65 (0.31)	63.44 (1.21)	<0.001
PIR	2.99 (0.04)	2.43 (0.15)	<0.001
BMI	28.46 (0.13)	29.60 (0.76)	0.109
HDL	1.37 (0.01)	1.39 (0.04)	0.627
TC	5.02 (0.02)	4.98 (0.09)	0.434
eGFR	97.63 (0.41)	75.94 (2.09)	<0.001
ALT	25.75 (0.33)	25.27 (2.20)	0.629
AST	25.22 (0.28)	25.63 (0.81)	0.05
SII	552.94 (6.41)	583.28 (23.81)	0.05
SIRI	1.23 (0.02)	1.56 (0.08)	<0.001
White blood cells			
Platelet (×10^3^ cells/ml)	254.86 (1.29)	238.9 (5.26)	0.005
Monocyte (×10^3^ cells/ml)	0.56 (0.01)	0.63 (0.02)	<0.001
Lymphocyte (×10^3^ cells/ml)	2.18 (0.03)	2.08 (0.14)	0.05
Neutrophils (×10^3^ cells/ml)	4.31 (0.02)	4.59 (0.08)	0.017
**Gender**			0.797
Women	50.46 (48.76 ,52.16)	51.72 (42.25 ,61.08)	
Men	49.54 (47.84 ,51.24)	48.28 (38.92 ,57.75)	
**Race**			0.003
Non-Hispanic white people	65.61 (63.19 ,67.95)	61.93 (52.89 ,70.21)	
Non-Hispanic black people	11.10 (9.93 ,12.39)	19.83 (14.40 ,26.67)	
Mexican American	9.55 (8.36 ,10.88)	7.71 (4.82 ,12.12)	
Other races	13.74 (12.39 ,15.23)	10.53 (6.11 ,17.54)	
**Education levels**			<0.001
Less than 9th grade	6.35 (5.66 ,7.13)	12.71 (8.92 ,17.80)	
9-11th grade/high school grade or equivalent	35.45 (33.59 ,37.36)	43.99 (34.92 ,53.48)	
College graduate or above	58.19 (56.04 ,60.31)	43.30 (33.90 ,53.21)	
**Diabetes mellitus**			<0.001
No	89.29 (88.21 ,90.28)	70.35 (62.43 ,77.21)	
Yes	10.71 (9.72 ,11.79)	29.65 (22.79 ,37.57)	
**Coronary heart disease**			<0.001
No	97.23 (96.48 ,97.81)	84.55 (77.84 ,89.50)	
Yes	2.77 (2.19 ,3.52)	15.45 (10.50 ,22.16)	
**Hyperlipidemia**			<0.001
No	32.58 (31.00 ,34.21)	17.22 (11.68 ,24.66)	
Yes	67.42 (65.79 ,69.00)	82.78 (75.34 ,88.32)	
**Hypertension**			<0.001
No	67.46 (65.77 ,69.10)	23.67 (14.34 ,36.47)	
Yes	32.54 (30.90 ,34.23)	76.33 (63.53 ,85.66)	
**Smoking**			0.132
non-Active smoker	78.90 (77.23 ,80.49)	73.17 (64.65 ,80.26)	
Active smoker	21.10 (19.51 ,22.77)	26.83 (19.74 ,35.35)	
**Alcohol use**			<0.001
non-Active alcohol user	33.98 (32.00 ,36.02)	57.83 (48.08 ,67.01)	
Active alcohol user	66.02 (63.98 ,68.00)	42.17 (32.99 ,51.92)	
**Antihypertensive medication**			<0.001
No	93.20 (92.26 ,94.04)	80.13 (72.24 ,86.21)	
Yes	6.80 (5.96 ,7.74)	19.87 (13.79 ,27.76)	
**Diabetes medications**			<0.001
No	93.39 (92.53 ,94.15)	78.82 (71.71 ,84.54)	
Yes	6.61 (5.85 ,7.47)	21.18 (15.46 ,28.29)	

Continuous variables are expressed as weighted mean (Standard error, SE).

Categorical variables are expressed as weighted % (95% confidence interval).

PIR, Poverty income ratio; BMI, body mass index. TC, Total cholesterol; HDL, high-density lipoprotein cholesterol; eGFR, estimated glomerular filtration rate; ALT, alanine aminotransferase; AST, aspartate aminotransferase.

### Correlation analysis of SII, SIRI with baseline characteristics

According to the Spearman rank correlation analysis, there was no significant correlation between SII levels and baseline characteristics (r<0.1). Similarly, SIRI levels were not significantly correlated with most baseline characteristic variables, except for a weak correlation observed with race (r=-0.103) (**Table S1**).

### Grouping based on SII and SIRI cut-off values

**Figure 2:**
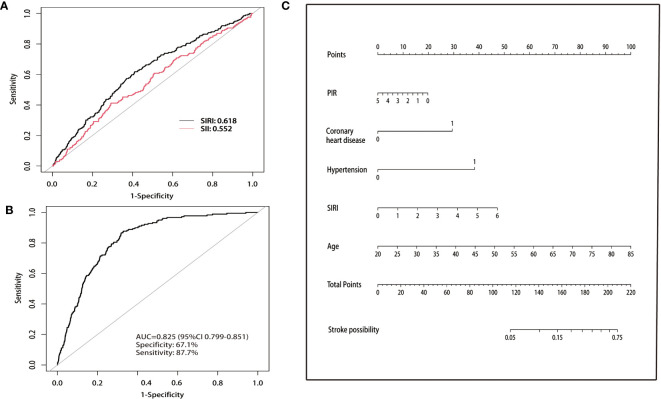
**(A)** Comparison of the predictive value of SII and SIRI for stroke prevalence. **(B)** Nomogram of ROC curves for predicting stroke prevalence in asthmatic populations. ROC, receiver operating characteristic. **(C)** Nomogram for predicting the probability of stroke prevalence. The top horizontal line is the score column, and the total score added item by item according to the score of each item corresponds to the probability of stroke in a patient with asthma. Age, SIRI, and PIR indicate the value of an individual. In the coronary artery disease and hypertension groups, 0 indicates “no” and 1 indicates “yes”. SIRI, systemic inflammation response index; PIR, poverty income ratio.

The ROC curve (**Figure 2**) was used to determine the optimal cut-off value for SII, SIRI, and stroke prevalence. The population was then divided into low and high SII groups based on the optimal cut-off at 609 (×10^3^ cells/μL). Similarly, the population was divided into low and high SIRI groups based on the optimal cut-off of 1.12 (×10^3^ cells/μL). Among patients with asthma, SIRI demonstrated superior predictive value (AUC=0.618) for stroke prevalence Compared with SII (AUC=0.552).

### Selection of adjustment variables

Candidate predictors that showed significant differences between the two groups at baseline were selected for the multivariate regression models. The unadjusted model did not include any covariates. Model 1 included adjustments for age and race, while model 2 included adjustments for age, race, PIR, aspartate aminotransferase (AST), eGFR, and education levels. Model 3 adjusted for age, race, PIR, AST, eGFR, education levels, diabetes mellitus, coronary heart disease, hyperlipidaemia, hypertension, alcohol use, antihypertensive medication, and diabetes medications.

### Univariate and multivariate logistic regression models

In **Table 2**, it can be observed that in the unadjusted model, the higher SII group had a higher prevalence of stroke compared with the lower SII group (OR 1.65; 95% CI, 1.17-2.34). This association persisted after adjusting for age and race in model 1 (OR 1.70; 95% CI, 1.18-2.45). After further adjustment for PIR, eGFR, AST, and education levels in model 2, a significant association between higher SII and higher stroke prevalence was observed (OR 1.77; 95% CI, 1.19-2.63). Furthermore, the association persisted after adjusting for all confounders in model 3 (OR 1.80; 95% CI, 1.18-2.76).

**Table 2:** Survey-weighted logistic regression examining the association of SII, SIRI with the prevalence of stroke among individuals with asthma from NHANES 1999 to 2018.

		Unadjusted Model	Adjusted	Adjusted	Adjusted
			Model 1	Model 2	Model 2
** **	**Cut-off point(×10^3^ cells/μL)**	**OR (95%CI)**	**OR (95%CI)**	**OR (95%CI)**	**OR (95%CI)**
**SII**	<609	Reference	Reference	Reference	Reference
	≥609	1.65 (1.17-2.34) ^a^	1.70 (1.18-2.45) ^a^	1.77 (1.19-2.63) ^a^	1.80 (1.18-2.76) ^a^
**SIRI**	<1.12	Reference	Reference	Reference	Reference
	≥1.12	3.0 (1.97-4.55) ^a^	2.75 (1.78-4.27) ^a^	2.45 (1.53-3.93) ^a^	2.23 (1.39-3.57) ^a^

Crude model adjusted no variables. ^a^ indicated the P-vale < 0.05.

Model 1 adjusted age, race.

Model 2 adjusted age, race, PIR, eGFR, AST, education levels.

Model 3 adjusted age, race, PIR, eGFR, AST, education levels, diabetes mellitus, coronary heart disease, hyperlipidemia, hypertension, alcohol use, antihypertensive medication, diabetes medications.

The findings for SIRI were comparable to those for SII, as higher SIRI was associated with a higher stroke prevalence in the unadjusted model (OR 3.0; 95% CI, 1.97-4.55). This association persisted even after adjusting for age and race in model 1 (OR 2.75; 95% CI, 1.78-4.27). Furthermore, in model 2, after additional adjustment for PIR, eGFR, AST, and education level, higher SIRI was still associated with higher stroke prevalence (OR 2.45; 95% CI, 1.53-3.93). Finally, in model 3, after adjusting for all confounders, higher SIRI remained associated with a higher stroke prevalence (OR 2.23; 95% CI, 1.39-3.57).

### Subgroup analysis

**Figure 3:**
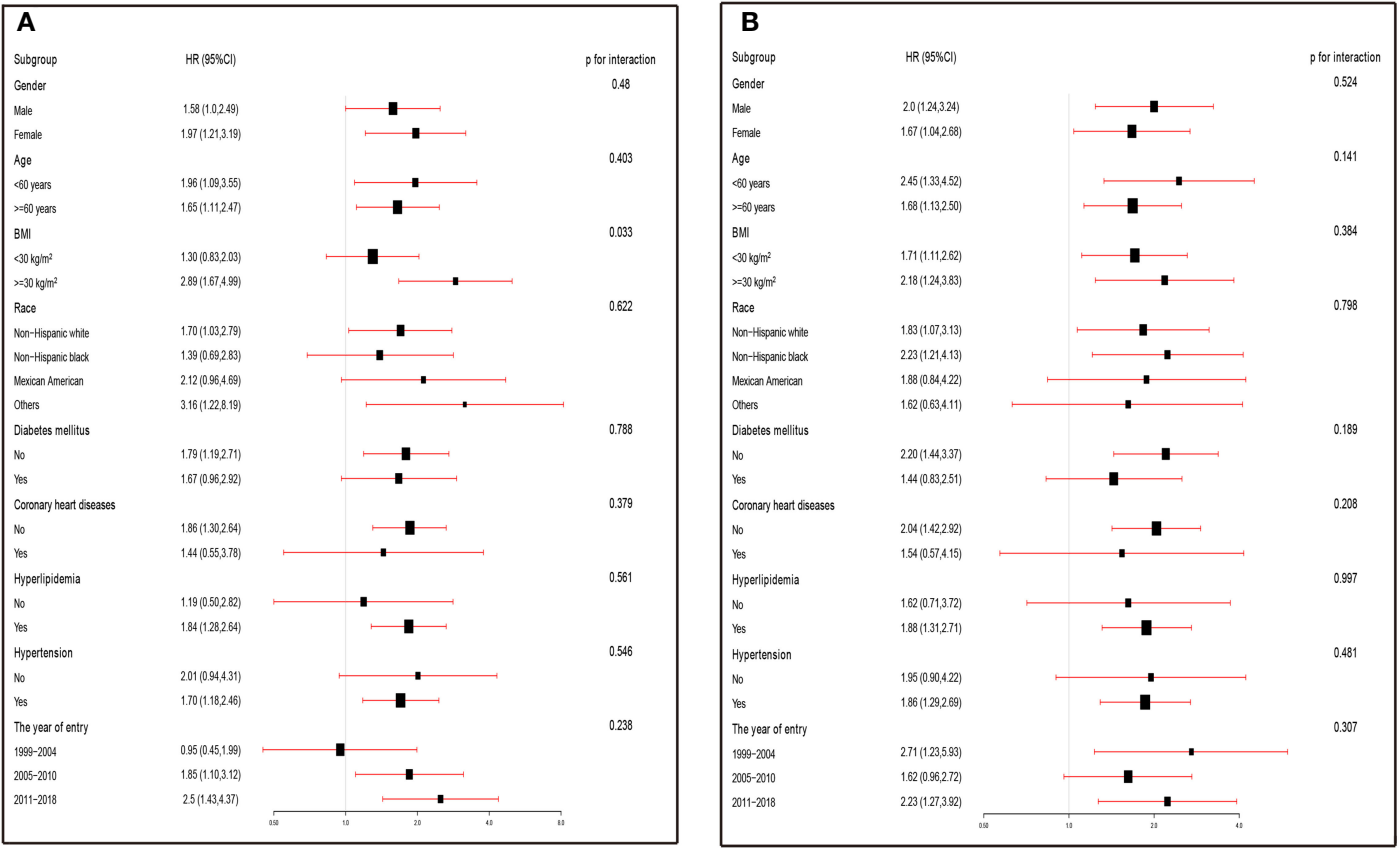
**(A)** Subgroup analysis was performed by comparing the risk of stroke prevalence (odds ratios, 95% CIs) in the high SII and low SII groups. The results were expressed as the risk of stroke prevalence in the high SII group compared with the low SII group in the different strata. **(B)** Subgroup analysis was performed by comparing the risk of stroke prevalence (odds ratios, 95% CIs) in the high SIRI and low SIRI groups. The results were expressed as the risk of stroke prevalence in the high SIRI group compared with the low SIRI group in the different strata. ORs were fully adjusted by the following covariates including age, race, PIR, eGFR, education levels, diabetes mellitus, coronary heart disease, hyperlipidemia, hypertension, alcohol use, antihypertensive medication, diabetes medications.

The findings in **Figure 3A** were consistent across all strata except for the BMI group (P=0.025), and no interaction was observed (P >0.05), indicating that the risk of stroke prevalence increased in asthmatics with higher SII levels in most strata. However, in certain subgroups (such as women, BMI<30 kg/m^2^, and those without diabetes mellitus, coronary heart disease, hypertension, and hyperlipidaemia), higher SII levels were more strongly associated with a higher prevalence of stroke, with an increased odds risk ranging from 68% to 189%. Similarly, in all strata, higher SIRI levels were associated with higher stroke prevalence, with no significant interaction observed (P>0.05). Consistent with the results for SII, higher SII levels were more closely associated with higher stroke prevalence in some specific populations (e.g., women, BMI ≥30 kg/m^2^, hyperlipidemia, absence of diabetes, coronary artery disease, and hypertension), with an increased odds risk ranging from 88% to 120% (**Figure 3B**).

### Construction of the nomogram

The multivariate logistic regression analysis revealed that five clinical variables (SIRI, age, PIR, coronary heart disease, and hypertension) were selected for the final clinical model construction, as presented in **Table S2**. The overall model displayed good predictive value with an AUC of 0.825, along with a sensitivity of 67.1% and specificity of 87.7%, as demonstrated in **Figure 2B**. Furthermore, the nomogram assigned a score to each clinical feature, which was summed up to obtain a total score, indicating the likelihood of stroke in a patient with asthma (**Figure 2C**).

## Discussion

This is the first study to investigate the association between SII, SIRI levels and the prevalence of stroke in an asthmatic population. The main findings of this study are as follows: 1) SII, SIRI were weakly correlated or not correlated with baseline clinical characteristics, indicating that they are a relatively stable inflammation indicator. 2) In the asthmatic population, higher SII and SIRI levels are strongly associated with a higher stroke prevalence. SIRI is an independent risk factor for stroke prevalence in patients with asthma, and its predictive value for stroke prevalence is superior to that of SII.3) 3). The clinical model consisting of age, SIRI, coronary heart disease, hypertension, and poverty income ratio has good predictive value and certain clinical significance.

The crucial role of chronic inflammation in stroke development is well-established, and asthma is known to be an inflammatory disease, implying that inflammation is a common background for both diseases (44–46). Meanwhile, there are some specificities of inflammation and stroke risk in asthmatics.1) Chronic inflammation: Asthma is a chronic inflammatory airway disease associated with systemic low-grade inflammation. Studies have proven that systemic inflammation may increase the risk of stroke (47, 48). 2) Airway inflammatory cells: Airway inflammation in asthmatics involves a variety of immune cells, such as eosinophils, lymphocytes, neutrophils and monocytes. Abnormal activation of these cells in asthmatic patients may be associated with the development and progression of atherosclerosis, thereby increasing the risk of stroke (49). 3) Inflammatory markers: Patients with asthma may have higher levels of inflammatory markers (such as C-reactive protein, IL-6), and these markers are associated with an increased risk of stroke (50, 51). 4) Other related factors: Patients with asthma may have other risk factors, such as obesity, hypertension, and hyperlipidemia. These factors may work together to increase the risk of stroke in patients with asthma. 5) Medication effects: Certain asthma medications, such as oral corticosteroids, may increase the cardiovascular risk and thus the risk of stroke. However, this risk may vary according to individual differences and type of medication. In summary, there is some specificity between inflammation and stroke risk in asthmatic patients. In clinical practice, a comprehensive assessment of asthmatic patients and control of associated risk factors are important strategies to reduce the risk of stroke.

In the present study, we observed that higher SII and SIRI levels were associated with a higher stroke prevalence in patients with asthma, even after adjusting for confounding variables. This suggests that a more intense inflammatory response is associated with stroke prevalence in patients with asthma. Furthermore, the association between SII and stroke prevalence was consistent across most subgroups, except for the BMI group, where the association was stronger. There is a strong correlation between SII, SIRI, and stroke prevalence in obese populations compared with non-obese populations. Obesity is strongly associated with the risk of stroke, and this association is independent of age, lifestyle, and other cardiovascular risk factors. Obesity is the accumulation of abnormal adipose tissue due to excessive calories and fat (52). Excessive adipocyte accumulation could result in various metabolic diseases, frequently accompanied by chronic low-grade, systemic, and local inflammation (53). The findings presented in **Table S3** suggest that obese populations have higher SII and SIRI levels. Similarly, the prevalence of stroke is more closely associated with SII and SIRI levels in the hyperlipidaemia subgroup. Hyperlipidaemia triggers inflammatory responses and promotes atherosclerosis formation (54). Cell activating factors and ILs, promote inflammation, enhance immune cell activity, increase platelet aggregation, damage endothelial cells, and accelerate atherosclerosis progression resulting in the onset of stroke (55). As presented in **Table S3**, higher SII and SIRI levels were observed in the hyperlipidaemia subgroup. Interestingly, although individuals with diabetes mellitus, coronary heart disease, and hypertension had higher SII and SIRI levels, their association with stroke was not further increased. The precise mechanism behind the phenomenon remains unclear, but it is likely associated with two aspects. First, unlike diabetes, coronary heart disease, hypertension obesity, and hyperlipidaemia are considered the “initiators” of inflammation. This is because obesity and hyperlipidaemia can directly trigger a systemic inflammatory response, whereas diabetes, coronary artery disease, and hypertension are the result of existing systemic inflammation. Second, the association between SII, SIRI, and stroke might be weakened by pharmacological interventions and primary prevention measures. Insulin resistance is closely associated with chronic inflammation, and the use of glucose-lowering drugs might ameliorate insulin resistance and decrease chronic inflammation (56). Medications such as statins and aspirin help reduce the inflammatory response in individuals with coronary artery disease (56, 57). Hypertension is often accompanied by a systemic inflammatory response characterised by the activation of complement, bone marrow, and vascular cell perturbations (58). However, blood pressure reduction might reverse these processes, and antihypertensive drugs such as angiotensin-converting enzyme inhibitors have been shown to decrease C-reactive protein, IL-6, and TNF-α levels (59).

### Perspectives

The relationship between inflammation and the development of asthma and stroke has been an area of research in the neuro-respiratory field. This study highlights the potential utility of SII and SIRI as robust inflammation markers, offering physicians a valuable tool to better comprehend the conditions and develop treatment plans while minimising the impact of confounding factors. Moreover, this study supports the strong association between elevated SII and SIRI levels and higher prevalence of stroke in patients with asthma, enabling physicians to identify disease risks earlier and take preventive measures. However, SIRI demonstrated greater predictive value than SII, and the clinical predictions generated by SIRI were found to be more effective.

### Limitations

To begin with, this cross-sectional study cannot establish a cause-and-effect relationship between SII levels and stroke prevalence. The diagnosis of asthma and stroke primarily relied on self-reported questionnaires, which might be less precise than medical diagnoses. Moreover, subjective self-reported asthma and stroke diagnoses with potential recall bias might have influenced the current findings. Second, although we have adequately adjusted for potential covariates in the regression analysis, the effect of residual variables (e.g., autoimmune and inflammatory diseases) on the results could not be ruled out. Third, the study was limited to participants from the United States; therefore, the results might not be generalisable to other populations with different risk factors and health behaviours. We divided the participants into three small intervals over time and then performed subgroup analyses to prevent treatment differences between generations from influencing the study results. The results showed no significant interactions between the intervals, indicating that treatment strategies across generations had a minor effect on the study outcomes. However, we still need to be aware that differences in treatment strategies across generations might have some influence on the current findings. Fourth, in the NHANES database, the medical history of participants is obtained primarily through standardised questionnaires during home follow-ups. We could not obtain further information on the specific typology of asthma or the current condition. Therefore, the impact of asthma phenotype and disease severity on the current outcome remains unknown. Given the study’s limitations, future research needs to further explore the roles of SII and SIRI in asthma and stroke prevalence. This will assist us in better understanding the common pathological mechanisms of asthma and stroke and provide more insights for the development of more effective treatment strategies.

## Conclusion

In asthmatics, higher levels of SII and SIRI significantly increase the prevalence of stroke, particularly in those with coexisting obesity and hyperlipidaemia. SII and SIRI are relatively stable novel markers of inflammation that are not significantly correlated with individual baseline characteristics or underlying diseases in individuals with asthma. Notably, SIRI exhibited a better predictive value for stroke prevalence compared with SII. Besides, the clinical model constructed with SIRI, age, coronary heart disease, diabetes mellitus, and poverty income ratio has good predictive value for stroke prevalence in the asthmatic population and holds certain clinical value.

## Data availability statement

The original contributions presented in the study are included in the article/**Supplementary Material**. Further inquiries can be directed to the corresponding author.

## Ethics statement

The studies involving human participants were reviewed and approved by National Center for Health Statistics Institutional Review Board. The patients/participants provided their written informed consent to participate in this study.

## Author contributions

WC designed this topic. WC drafted, analyzed, and interpreted this study. WC, XB, CX, GW, FK, HP, SY, and SC critically reviewed the study. All authors contributed to the article and approved the submitted version.
