# Supplementary material for: Higher systemic immune-inflammation index and systemic inflammation response index levels are associated with stroke prevalence in the asthmatic population: a cross-sectional analysis of the NHANES 1999-2018
Source: Front Immunol. 2023 Aug 4;14:1191130. doi: 10.3389/fimmu.2023.1191130 (PMC10436559; doi:10.3389/fimmu.2023.1191130)
Supplement: Supplementary file 1 [file Table_1.pdf]

Table S1. Spearman correlation analysis of SII, SIRI and baseline characteristics							
		**Correlation(r)**	**P-value**			**Correlation(r)**	**P-value**
**SII**	**Age**	-0.021	0.1	**SIRI**	**Age**	0.046	<0.001
	**Gender**	-0.073	<0.001		**Gender**	0.115	<0.001
	**Race**	-0.051	<0.001		**Race**	-0.103	<0.001
	**Education levels**	-0.004	0.775		**Education levels**	-0.021	0.128
	**PIR**	-0.008	0.561		**PIR**	-0.021	0.126
	**BMI**	0.081	<0.001		**BMI**	0.067	<0.001
	**TC**	0.007	0.611		**TC**	-0.067	<0.001
	**HDL**	-0.036	0.007		**HDL**	-0.116	<0.001
	**ALT**	-0.037	0.005		**ALT**	0.032	0.154
	**AST**	-0.092	<0.001		**AST**	-0.006	0.628
	**eGFR**	-0.001	<0.001		**eGFR**	-0.093	<0.001
	**CHD**	-0.001	0.937		**CHD**	0.082	<0.001
	**DM**	0.016	0.207		**DM**	0.044	<0.001
	**Hyperlipidemia**	0.060	<0.001		**Hyperlipidemia**	0.050	<0.001
	**Hypertension**	0.022	0.088		**Hypertension**	0.065	<0.001
	**Smoking**	0.184	0.158		**Smoking**	0.068	<0.001
	**Alcohol use**	-0.013	0.316		**Alcohol use**	-0.002	0.885
	**Antihypertensives**	0.008	0.540		**Antihypertensives**	0.019	0.150
	**Diabetes medications**	0.030	0.023		**Diabetes medications**	0.050	<0.001

PIR, Poverty income ratio. BMI, Body mass index. ALT, Alanine aminotransferase. AST, Aspartate aminotransferase.TC, Total cholesterol. HDL, High-density lipoprotein cholesterol. eGFR, estimated glomerular filtration rate. DM, diabetes mellitus. CHD, coronary heart disease. SII, Systemic immune-inflammation index. SIRI, systemic inflammation response index.

Table S2. Variables were screened by multivariate logistic regression models to construct clinical models.

	OR	95%CI low	95%CI upper	P-value
**SII**	0.99953	0.99881	1.00025	0.202743
**SIRI**	1.32522	1.03870	1.69079	0.025043
**Age**	1.04843	1.02993	1.06726	<0.000001
**PIR**	0.84760	0.73008	0.98403	0.031607
**eGFR**	0.99789	0.98769	1.00820	0.687754
**AST**	1.00296	0.99802	1.00793	0.243089
**Race**	1.04156	0.87137	1.24497	0.655347
**Education levels**	0.95927	0.67911	1.35501	0.813803
**Diabetes mellitus**	0.92245	0.50091	1.69874	0.795943
**Hyperlipidemia**	1.22277	0.70783	2.11230	0.472082
**Hypertension**	3.06583	1.50714	6.23653	0.002404
**Coronary heart disease**	1.95520	1.11290	3.43502	0.021146
**Alcohol use**	0.81673	0.54257	1.22944	0.333681
**Antihypertensive medication**	0.94245	0.53935	1.64680	0.835401
**Diabetes medications**	1.52575	0.76938	3.02571	0.228550

*In the multivariate logistic regression model, SII and SIRI were analyzed as continuous variables.

Table S3. Differences in SII and SIRI levels between subgroups

**BMI**	**<30kg/m^2**	**≥30kg/m^2**	**P-values**
SII	537.45 (8.34)	582.11 (9.01)	0.001
SIRI	1.20 (0.02)	1.29 (0.02)	0.006
**Coronary heart disease**	**No**	**Yes**	
SII	548.72 (6.64)	592.84 (17.49)	0.02
SIRI	1.22 (0.02)	1.36 (0.02)	0.003
**Hyperlipidemia**	**No**	**Yes**	
SII	528.59 (10.11)	565.57 (8.24)	0.007
SIRI	1.19 (0.03)	1.25 (0.02)	0.07
**Hypertension**	**No**	**Yes**	
SII	538.91 (8.18)	582.81 (9.4)	<0.001
SIRI	1.18 (0.02)	1.34 (0.03)	<0.001
**Diabetes mellitus**	**No**	**Yes**	
SII	548.72 (6.64)	592.84 (17.49)	0.02
SIRI	1.22 (0.02)	1.36 (0.05)	0.003
